# Isolation of bioactive compounds from the petroleum ether soluble fraction of *Eichhornia crassipes* (Mart.) Solms flowers with dual evaluation: *In silico* studies of isolated molecules and *in vitro*/*in vivo* activities of the extract

**DOI:** 10.1371/journal.pone.0351085

**Published:** 2026-06-08

**Authors:** Md. Mirazul Islam, Suriya Akter Shompa, Hasin Hasnat, Safaet Alam, Saima Jahan Riti, Ferdousy Kabir, Md. Sakhawat Hossain, Mohammad A. Rashid

**Affiliations:** 1 Department of Pharmacy, School of Pharmaceutical Sciences, State University of Bangladesh, Dhaka, Bangladesh; 2 Department of Pharmaceutical Chemistry, Faculty of Pharmacy, University of Dhaka, Dhaka, Bangladesh; 3 Chemical Research Division, BCSIR Dhaka Laboratories, Bangladesh Council of Scientific and Industrial Research (BCSIR), Dhaka, Bangladesh; 4 Pharmaceutical Sciences Research Division, BCSIR Dhaka Laboratories, Bangladesh Council of Scientific and Industrial Research (BCSIR), Dhaka, Bangladesh; University of Sahiwal, PAKISTAN

## Abstract

*Eichhornia crassipes* (water hyacinth), an invasive aquatic plant, has attracted interest as a potential source of pharmacologically active natural products despite its ecological impact. The present study investigated the petroleum ether soluble fraction (PSF) of the methanolic extract of E. crassipes flowers to identify bioactive constituents and evaluate their biological activities. Chromatographic isolation followed by ¹H NMR spectroscopy led to the identification of five compounds: Kaempferol (Compound 1), Luteolin (Compound 2), Stigmasterol (Compound 3), 4-carboxybenzyl alcohol (Compound 4), and 4-methoxybenzaldehyde (Compound 5). Antimicrobial activity was assessed using disc diffusion and minimum inhibitory concentration (MIC) assays, thrombolytic activity through an in vitro clot lysis method, and antidiarrheal activity using the castor oil–induced diarrhea model in mice. Molecular docking and ADMET analyses were performed to explore potential target interactions and pharmacokinetic characteristics. The PSF exhibited moderate antimicrobial activity, with the highest inhibition against *Bacillus subtilis* (20.33 ± 1.25 mm) and MIC values ranging from 15.6–500 μL/mL. The extract also produced 14% clot lysis in the thrombolytic assay. *In vivo* antidiarrheal testing showed dose-dependent inhibition, reaching 38.89% reduction in diarrheal count at 600 mg/kg, approaching the activity of loperamide. Molecular docking analysis revealed that the identified compounds exhibited potential binding affinities comparable to standard ligands, suggesting potential interactions with relevant biological targets. The stigmasterol showing the highest interaction toward the κ-opioid receptor (−10.3 kcal/mol), comparable to loperamide (−9.1 kcal/mol). Flavonoids such as kaempferol and luteolin demonstrated notable binding with antimicrobial targets including dihydrofolate reductase (DHFR) and β-ketoacyl-ACP synthase (KAS), as well as thrombolytic target tissue plasminogen activator (TPA). ADMET predictions indicated favorable drug-likeness for the flavonoids but highlighted lipophilicity-related limitations for stigmasterol. Collectively, these findings highlight the PSF of *E. crassipes* as a promising reservoir of multi-target phytochemicals with potential applications in gastrointestinal, infectious, and thromboembolic disorders.

## Introduction

For centuries, plants have served as a fundamental source of therapeutic agents, playing a pivotal role in global healthcare [1]. The World Health Organization reports that nearly 80% of the world’s population relies on traditional medicine, highlighting the continued relevance of plant-based treatments [2]. Compared to synthetic drugs, plant-derived medicines are often associated with therapeutic efficacy and reduced adverse effects, which supports their widespread use in traditional systems such as Ayurveda and Chinese medicine [3]. These systems utilize diverse bioactive compounds that continue to attract attention for the development of novel therapeutics [4]. Projections further suggest that plant-based therapies may constitute a substantial proportion of medicines, particularly in developing regions [5]. In the modern era, medicinal plants remain a promising source for managing conditions such as pain, oxidative stress, cancer, and other chronic diseases, owing to their accessibility, cost-effectiveness, and therapeutic potential [3].

*Eichhornia crassipes*, or water hyacinth, is an invasive aquatic plant native to South America’s Amazon basin [6]. It has spread globally, adapting to various climates and aquatic environments, from lakes to rivers, disrupting ecological balance. The plant is characterized by its round to oval leaves, covered petioles, and blue or lavender flowers. Its air-filled sacs enable it to float on water, while its extensive root system facilitates nutrient absorption [6,7]. Despite its ecological impact, water hyacinth has been utilized in traditional medicine worldwide. In Chinese medicine, it is used to support spleen health, while in the Philippines, it serves as an anti-inflammatory agent. In Kenya, it aids lactation and menstrual regulation, and its beans are consumed to alleviate digestive issues. Modern research has identified its potential anti-cancer properties, alongside its antifungal, antibacterial, and wound-healing abilities [2,8].

Taxonomically, water hyacinth belongs to the Pontederiaceae family and is rich in bioactive compounds such as alkaloids, flavonoids, and terpenoids. These compounds contribute to its neuropharmacological effects, including analgesic, anti-epileptic, and memory-enhancing properties [2]. The plant also exhibits hepatoprotective, antioxidant, and antitumor activities, making it a valuable resource for pharmaceutical and cosmetic applications. Its ability to absorb heavy metals further underscores its utility in phytoremediation. The diverse phytochemical composition of water hyacinth supports its use in treating gastrointestinal disorders, inflammation, and pain, highlighting its significance in both traditional and modern medicine [9,10].

Global health issues, including diarrheal diseases and the rise of antimicrobial resistance (AMR), highlight the critical need for innovative therapeutic solutions. Diarrheal diseases remain a major global health concern, contributing to approximately 1.5 million deaths annually worldwide, with recent estimates from the Global Burden of Disease (GBD 2021) study indicating that this burden remains substantial, particularly in low- and middle-income countries [11,12]. One of the main causes of diarrheal mortality is poor sanitation and infections caused by pathogens such as *Candida albicans, Escherichia coli*, and *Salmonella typhi* [13,14]. In addition, antimicrobial resistance represents a major global health threat, and projections suggest that AMR could cause up to 10 million deaths per year by 2050 if effective interventions are not implemented [15]. Although a wide range of synthetic drugs is available for treating infections, these often come with substantial side effects [2]. On the other hand, plant-based medicines are increasingly recognized for their enhanced therapeutic efficacy and reduced adverse effects [16]. Resulting increased interest in plant-based antimicrobial agents as potential alternatives to traditional antibiotics for tackling these pressing health concerns. Consequently, the development of new antibacterial agents derived from natural sources holds promise in controlling bacterial proliferation and addressing the issue of antimicrobial resistance [1]. Natural plant-based compounds have recently emerged as a valuable reservoir of novel, safe, and potent bioactive metabolites with significant therapeutic potential.

This study aimed to investigate the phytochemical composition and pharmacological potential of *Eichhornia crassipes* flower extracts through an integrated analytical and biological approach. Chromatographic separation combined with ¹H NMR spectroscopy was employed to identify the major phytochemical constituents present in the petroleum ether soluble fraction. The biological activities of the extract were subsequently evaluated using in vitro antimicrobial and thrombolytic assays, along with *in vivo* antidiarrheal experiments. To explore possible molecular mechanisms underlying these activities, molecular docking analysis was performed to predict potential interactions between the identified compounds and selected biological targets. This computational approach was used to generate hypothesis-supporting insights rather than definitive mechanistic conclusions. By integrating phytochemical characterization with experimental pharmacological evaluation and predictive *in silico* analysis, the study provides a comprehensive assessment of the bioactive potential of *E. crassipes* flowers, while highlighting the need for further mechanistic and pharmacological validation.

## Materials and methods

### Drugs and chemicals

High-purity analytical-grade drugs and chemicals were utilized in this study. Methanol, Petroleum Ether, Dichloromethane, Ethyl Acetate and Tween-80 were obtained from Merck (Darmstadt, Germany). Loperamide, Streptokinase, Azithromycin, Amoxicillin, Ciprofloxacin, and Fluconazole were procured from Square Pharmaceuticals Ltd. and Beximco Pharmaceuticals Ltd, both located in Bangladesh.

### Microorganism

For the antimicrobial evaluation, a range of microorganisms was used, including gram-positive bacteria (*Sarcina lutea*, *Bacillus megaterium*, *Staphylococcus aureus*, *Bacillus cereus*, and *Bacillus subtilis*), gram-negative bacteria (*Vibrio mimicus*, *Pseudomonas aeruginosa*, *Salmonella typhi*, *Salmonella paratyphi*, *Escherichia coli*, *Shigella dysenteriae*, and *Vibrio parahaemolyticus*), and fungal strains (*Aspergillus niger*, *Saccharomyces cerevisiae*, and *Candida albicans*). These microbial strains were obtained from the University of Dhaka, Bangladesh.

### Plant collection

On November 11, 2022, the flowers of *Eichhornia crassipes* (Mart.) Solms were gathered from the Hawor region of Austagram, Kishoreganj, Dhaka, Bangladesh. The plant specimen was verified by a botanist at the Bangladesh National Herbarium on December 6, 2022, and given the unique accession number DACB87254. The plant material was then processed and stored in the phytochemical laboratory of the School of Pharmacy at the State University of Bangladesh for experimental purposes.

### Extraction process

The flower of *E. crassipes* was thoroughly cleaned and shed dried until the moisture content was reduced to around 10%. The dried plant was then ground into a coarse powder using a grinding machine. Approximately 800 grams of the powdered flowers were placed in a 5-liter round-bottomed flask, and 2.5 liters of methanol were added. The mixture was allowed to macerate for 21 days, with regular shaking and stirring. After maceration, the mixture (32% w/v) was filtered through Whatman No. 1 filter paper and a fresh cotton plug. This extraction process was repeated three times using analytical-grade ethanol. The combined filtrate was concentrated using a Buchi Rotavapor under reduced temperature and pressure, resulting in 83.5 grams of crude extract (10.43% yield).

To fractionate the crude extract, a modified Kupchan partitioning method was employed following established protocols [17]. Briefly, 50 g of the concentrated methanolic crude extract was suspended in 400 mL of distilled water:methanol (9:1, v/v) to ensure proper dissolution. The suspension was then sequentially partitioned with solvents of increasing polarity using a separatory funnel. Initially, the aqueous methanolic solution was extracted with petroleum ether (3 × 300 mL) to obtain the petroleum ether soluble fraction (PSF), targeting non-polar constituents such as lipids, sterols, and terpenoids. The remaining aqueous phase was subsequently partitioned with dichloromethane (3 × 300 mL) to yield the dichloromethane soluble fraction (DSF), followed by extraction with ethyl acetate (3 × 300 mL) to obtain the ethyl acetate soluble fraction (ESF). The final aqueous layer constituted the aqueous soluble fraction (ASF). Each fraction was separately concentrated under reduced pressure using a rotary evaporator at controlled temperature to yield PSF (16.1 g; 32.20% w/w), DSF (11.12 g; 22.24% w/w), ESF (12.18 g; 24.36% w/w), and ASF (7.32 g; 14.64% w/w). This systematic solvent partitioning enables efficient separation of phytoconstituents based on polarity, enhancing downstream biological and chemical analyses.

### Compound isolation procedure

The PSF of the extract was utilized for compound separation. Gel permeation chromatography (GPC/SEC) was performed using Sephadex LH-20 (Sigma-Aldrich), and preparative thin-layer chromatography (PTLC) and thin-layer chromatography (TLC) were carried out on silica gel 60 F254 aluminum sheets (Merck, Germany) with a thickness of 0.25 mm. The TLC plates were examined under a UV lamp (UVGL-58, United States) at 254 nm and 365 nm. For visualization, the plates were sprayed with a vanillin-sulfuric acid solution and heated at 100°C for 5 minutes. Pure compounds were isolated via the PTLC method, and their purity was verified using spot TLC.

### Compound identification procedure

The isolated compounds were subsequently analyzed using ¹H NMR spectroscopy. The spectra were recorded using Bruker spectrometers operating at 600 MHz. Samples were dissolved in deuterated solvents (CDCl_3_ or MeOD) depending on solubility, and chemical shifts (δ) were reported in ppm relative to tetramethylsilane (TMS) or residual solvent peaks as internal standards [18]. Spectral acquisition parameters included typical settings such as 32–2000 scans, relaxation delays (D1 = 1–2 s), and spectral widths ranging from approximately 11,900–32,600 Hz, ensuring adequate signal resolution and sensitivity. For instance, high-resolution spectra were obtained on a Bruker 600 MHz instrument using CDCl_3_ with 2000 scans and a relaxation delay of 2 s, while MeOD-based spectra were recorded with 64 scans and a relaxation delay of 1 s. All spectra were processed using exponential line broadening (LB = 0.30–1.0 Hz), followed by baseline correction and phase adjustment prior to integration. Integration was performed on baseline-corrected spectra to ensure accurate proton quantification, and peak assignments were made based on chemical shift, multiplicity, and comparison with published literature data.

### *In vitro* activities

#### Antimicrobial activities.

**Disc diffusion (zone of inhibition) assay:** The antimicrobial activity of the four fractions derived from the methanolic extract of *E. crassipes* flower was evaluated using the disc diffusion method [19]. Sterilized filter paper discs (6 mm diameter) impregnated with 100 µg of each test sample (CME, PSF, DSF, ESF, ASF) were placed onto nutrient agar plates pre-inoculated with bacterial and fungal strains. Positive controls included commercial antibiotic discs of Azithromycin, Amoxicillin, and Ciprofloxacin (30 µg/disc) and an antifungal disc of Fluconazole (30 µg/disc), while blank discs served as negative controls. The plates were inverted and stored at 4°C for 24 hours to allow even diffusion, followed by incubation at 37°C for an additional 24 hours. The zones of inhibition, reflecting antibacterial activity, were measured in millimeters [20]. The assay was conducted on clinically isolated strains of gram-positive bacteria (*Sarcina lutea*, *Bacillus megaterium*, *Staphylococcus aureus*, *Bacillus subtilis*, *Bacillus cereus*), gram-negative bacteria (*Vibrio mimicus*, *Pseudomonas aeruginosa*, *Salmonella typhi*, *Salmonella paratyphi*, *Escherichia coli*, *Shigella dysenteriae*, *Vibrio parahaemolyticus*), and fungal strains (*Aspergillus niger*, *Saccharomyces cerevisiae*, *Candida albicans*).

**Minimum inhibitory concentration (MIC) assay:** The minimum inhibitory concentration (MIC) of the extracts and fractions was determined against the tested microorganisms using a serial dilution method in a microtiter plate [21,22]. Initially, the test samples were dissolved in dimethyl sulfoxide (DMSO) to obtain a stock solution of 500 μL/mL. Subsequently, two-fold serial dilutions of the samples were prepared in sterile nutrient broth within the wells of a 96-well microtiter plate to obtain a range of decreasing concentrations. A microbial suspension was prepared and adjusted to an optical density of 0.01–0.1 at 600 nm, corresponding to approximately 10⁷ cells/mL. Then, 10 μL of the microbial inoculum was added to each well containing 100 μL of sterile growth medium and the diluted test samples. The microtiter plates were incubated at 37 °C for 24 h for bacterial strains and at 25 °C for 48–72 h for fungal strains. After incubation, the wells were visually examined for microbial growth. The lowest concentration of the test sample that showed no visible growth of microorganisms was recorded as the MIC value, expressed in μL/mL.

#### Thrombolytic assay.

The thrombolytic potential of the CME, PSF, DSF, ESF, and ASF of *E.crassipes* flower was assessed using streptokinase (SK) as the reference standard, following the protocol established by Prasad et al. (2006) [23]. Venous blood was collected from a healthy volunteer after obtaining written informed consent prior to sample collection. The experimental procedure was conducted in accordance with the ethical guidelines approved by the Institutional Ethics Review Committee of the State University of Bangladesh (Approval No. **2023-10-30/SUB/I-ERC/003**). The blood sample was collected on 10 Nov 2023. Six Eppendorf tubes were weighed, and 500 µL of blood was added to each tube. The tubes were incubated at 37°C for 45 minutes to allow clot formation. After coagulation, the serum was removed, and the tubes were reweighed to determine the clot weight. Subsequently, 100 µL of each fraction and the standard streptokinase solution were added to the respective tubes. The tubes were incubated again at 37°C for 90 minutes to promote clot lysis. After incubation, the released fluid was removed, and the tubes were weighed again to measure the weight change resulting from clot disruption. The percentage of clot lysis was calculated based on the weight difference before and after the lysis process.

The percentage of clot lysis was calculated using the following formula:


$$\begin{array}{c} \%\text{ Clot Lysis }=\frac{\text{Weight of the Lysed Clot}}{\text{Weight of the Clot Before Lysis}}\times \text{1}\text{0}\text{0}\;\% \end{array}$$

### *In vivo* analysis

#### Animal preparation.

For the *in vivo* study, total 25 Swiss white mice of both sexes, aged four to five weeks, were procured from the Animal Resource Branch of the International Centre for Diarrheal Diseases and Research, Bangladesh (ICDDR, B). The mice were housed in polypropylene cages under controlled environmental conditions, including a 12-hour light-dark cycle, a temperature of 24 ± 2°C, and a relative humidity of 60–70%. They were fed ICDDR, B-formulated rodent food and provided water ad libitum. To ensure proper acclimatization and reduce stress, the mice were maintained under these conditions for at least four days before the experiments. All procedures adhered to ethical guidelines and regulations for the care and use of laboratory animals. The study followed the approved Animal Use Protocol from the Ethics Committee, in compliance with the US National Institutes of Health guidelines for the Care and Use of Laboratory Animals. Recommendations from the Federation of European Laboratory Animal Science Associations (FELASA) were also followed to minimize animal pain and stress.

The number of animals used in each experimental group (n = 4) was selected based on commonly applied group sizes in preliminary pharmacological screening studies, where the objective is to identify potential biological activity prior to more extensive confirmatory investigations. This approach also aligns with the ethical principle of Reduction within the 3R framework (Replacement, Reduction, and Refinement) for responsible animal use.

Mice were monitored for signs of pain or distress, including >20% body-weight loss, severe lethargy, inability to access food or water, unresponsive behavior, persistent piloerection, impaired mobility, labored breathing, convulsions, or abnormal posture. Any animal exhibiting these signs was immediately removed from the study and humanely euthanized using an intraperitoneal overdose of anesthesia (Ketamine HCl at 100 mg/kg and Xylazine at 7.5 mg/kg), as per established protocols [24,25].

Animals were observed every 4 hours during the first 24 hours after treatment and twice daily thereafter for changes in general behavior, food and water intake, posture, and locomotor activity. Detailed monitoring records were maintained throughout the experimental period to document any clinical signs of distress or abnormal physiological responses. No animals died during the study, and none reached the predefined humane endpoint criteria. The study protocol was approved by the Animal Ethics Committee of the State University of Bangladesh, Dhaka (**2023-10-30/SUB/A-ERC/005**). All personnel involved in animal handling and monitoring were trained in laboratory animal care, recognition of distress indicators, and approved euthanasia procedures. Following completion of the study, all surviving animals were monitored twice daily for an additional two months, during which no abnormal clinical signs, behavioral changes, or health complications were observed; the animals subsequently returned to their normal housing environment without any adverse outcomes. These observations further confirmed that the experimental procedures did not induce long-term physiological or behavioral distress in the animals.

#### Acute oral toxicity test.

The methanol-soluble crude extract of *E. crassipes flower* was orally administered to mice at a high dose of 2000 mg/kg under controlled laboratory conditions, adhering to the fixed-dose method specified in OECD Guidelines 420 [4]. Throughout the acute toxicity assessment, animals were evaluated according to the same humane endpoints and monitoring schedule described above. During the 72-hour monitoring period, no allergic reactions, behavioral alterations (e.g., sedation or excitability), or fatalities were recorded. Consequently, doses of 200, 400 and 600 mg/kg (body weight, orally) were deemed safe and chosen for subsequent antidiarrheal activity evaluations.

#### Antidiarrheal assay.

The antidiarrheal activity of various fractions of *E. crassipes* was evaluated using the castor oil-induced diarrhea model in mice, following established protocols [26]. Diarrhea was induced in each mouse by administering 1 mL of high-purity analytical-grade castor oil, and the total number of fecal excretions was recorded. The mice were divided into five groups: control, positive control, and three test groups, each consisting of four mice. The control group received 10 mL/kg of 1% Tween 80 in water orally, while the positive control group was given 5 mg/kg of loperamide orally. The test groups were administered *E. crassipes* extract fractions at doses of 200, 400 and 600 mg/kg body weight respectively. After treatment, diarrhea was induced with castor oil, and each mouse was placed in an individual cage with hourly changes of the cage lining. The antidiarrheal effect was determined by comparing the test groups to the control group, with fecal counts recorded over a 4-hour observation period. The percentage inhibition of diarrhea was calculated using the formula:


$$\begin{array}{c} \%\text{ Inhibition of Defecation}=\frac{\text{Mean Defecation by Control}-\text{Mean Defecation by Test Samples or Standard}}{\text{Mean Defecation by Control}}\times \text{1}\text{0}\text{0}\;\% \end{array}$$

#### Statistical analysis.

The data were analyzed using GraphPad Prism 5.2 (GraphPad Software, Inc., La Jolla, CA, USA), and the results from the *in vivo* antidiarrheal experiments were expressed as mean ± standard error of the mean (SEM). Statistical significance was determined using one-way analysis of variance (ANOVA) and Dunnett’s test. Significance levels were marked as *p < 0.05, **p < 0.01, and ***p < 0.001 [4].

### Molecular docking

#### Software.

Computational docking studies were performed on the metabolites identified from *C. affinis* whole plant extracts using established software tools, including PyRx, PyMoL 2.3, Discovery Studio 4.5, and Swiss PDB viewer [27].

#### Ligand preparation.

For ligand preparation, the identified phytochemicals were retrieved from the PubChem database (https://pubchem.ncbi.nlm.nih.gov/), and their 3D structures were downloaded in SDF format. The 3D structures of reference compounds—Ciprofloxacin, amoxicillin, Loperamide, and Streptokinase—were also obtained. These ligands were imported into Discovery Studio 4.5, and the PM6 semi-empirical method was applied to optimize their structures for improved docking precision [28].

#### Target preparation.

For receptor preparation, the 3D crystal structures of target proteins were sourced from the Protein Data Bank (RCSB PDB, https://www.rcsb.org) [29]. The selection of target proteins followed a rational design strategy based on the pharmacological activities evaluated experimentally in this study. For antidiarrheal activity, the kappa-opioid receptor (KOR) [PDB: 6VI4] [30] and human delta-opioid receptor (DOR) [PDB: 4RWD] [31] were selected because opioid receptors play important roles in regulating intestinal motility and secretion. For antimicrobial analysis, β-ketoacyl-ACP synthase 3 (KAS) [PDB: 1HNJ] [32] and Dihydrofolate reductase (DHFR) [PDB: 4M6J] [33] were chosen due to their essential involvement in bacterial fatty acid biosynthesis and folate metabolism, respectively, making them well-established antimicrobial drug targets. To explore thrombolytic potential, tissue plasminogen activator (TPA) [PDB: 1A5H] [29] was used because of its key role in fibrinolysis and clot degradation. The protein structures were processed in PyMOL 2.3 to remove water molecules and ligands/residues, and non-polar hydrogen atoms were added. Energy minimization was performed using Swiss PDB viewer [34]. Ligand-receptor interactions were analyzed using PyRx Autodock Vina, employing semiflexible docking methods. Proteins were prepared as macromolecules, and specific amino acids were selected to ensure precise ligand binding to the target sites [5]. The selection of target site and grid mapping of target receptors are shown as Table 1.

**Table 1:** Selection of target site and grid mapping of target receptors for molecular docking of isolated compounds from PSF of methanolic extract of *E. crassipes* flower.

Target Activity	Receptor	Standard	Target binding sites	Reference	Grid box	
Antimicrobial	Dihydrofolate Reductase (DHFR) [PDB ID4M6J]	Ciprofloxacin (PubChem ID: 2764)	Asp 9, Phe 16, Ile 54, Gly 55, Ser 56, Ala 75, Lys 76, Glu 77, Leu 78, Tyr 91, Ile 92, Gly 93, Arg 117, Thr 118, Gln 119, Asp 120	[34]	Center	x = 3.30050538898
						y = −3.54749560527
						z = −18.6603468425
					Dimension	x = 20.8847111475
						y = 28.2427927367
						z = 27.1639553048
	Beta-ketoacyl-ACP synthases (KAS) [PDB ID:1HNJ]	Amoxicillin (PubChem ID: 33613)	Trp 32, Arg 36, Thr 37, Thr 81, Ala 109, Ala 110, Ala 111, Cys 112, Leu 142, Gly 152, Ile 155, Ile 156, Phe 157, Leu 189, Thr 190, Leu 191, Leu 205, Met 207, Gly 209, Asn 210, Val 212, Phe 213, Ala 216, Leu 220, His 244, Ala 246, Asn 247, Ile 250, Asn 274, Glu 302, Ala 303, Phe 304, Gly 306, Gly307	[4]	Center	x = 29.0033635958
						y = 17.3175380047
						z = 31.5701285056
					Dimension	x = 26.5571751807
						y = 35.4261745701
						z = 23.0503334713
Antidiarrheal	Human Delta-Opioid Receptor (DOR) [PDB ID: 4RWD]	Loperamide (PubChem ID: 3955)	A chain- Val 62, Leu 65, Gly 66, Leu 69, Val 70, Phe 72, Gly 73, Tyr 77, Pro 315, Val 316, Ala 319, Phe 325, Cys 328, Phe 329, Gln 331, Leu 332	[5]	Center	x = −56.6815476866
						y = 1.83509476912
						z = 52.8777794668
					Dimension	x = 21.9977639041
						y = 18.6802030241
						z = 21.1327889808
	Kappa Opioid Receptor (KOR) [PDB ID: 6VI4]		B chain- Leu 103, Leu 107, Ser 136, Ile 137, Try 140, Ile 180, Trp 183, Leu 184, Ser 187, Ile 191, Leu 192 Ile 194, Val 195	[29]	Center	x = 54.4225497657
						y = −50.4523701153
						z = −16.3056618844
					Dimension	x = 16.2392828969
						y = 28.2473102176
						z = 18.0419317341
Thrombolytic	Tissue Plasminogen Activator (TPA) [PDB ID: 1A5H]	Streptokinase (PubChem ID: 482240142)	Arg 39, Leu 41, Cys 42, His 57, Cys 58, Gln 60, Glu 60A, Phe 60C, Tyr 99, Tyr 151, Asp 189, Ala 190, Cys 191, Gln 192, Gly 193, Ser 195, Ile 213, Ser 214, Trp 215, Gly 216, Leu 217, Gly 219, Val 224, Pro 225, Gly 226, Val 227, and Tyr 228	[29]	Center	x = 5.31220314201
						y = 35.0614579238
						z = 49.2471129809
					Dimension	x = 27.1580470485
						y = 24.29842386
						z = 30.4915813269

#### ADME/T analysis.

In current drug discovery, computational methods that assess pharmacokinetic properties—absorption, distribution, metabolism, excretion, and toxicity (ADMET)—play a significant role in evaluating drug-like characteristics and bioavailability. These analyses are indispensable for exploring the pharmacological potential of new compounds (http://biosig.unimelb.edu.au/pkcsm/prediction). The Swiss ADME web tool (http://www.sib.swiss) was used to predict drug-likeness, applying Lipinski’s rules and pharmacokinetic parameters. Lipinski’s criteria state that a compound is likely to be orally active if it adheres to the following guidelines: a molecular weight below 500 atomic mass units (amu), up to 5 hydrogen bond donors, up to 10 hydrogen bond acceptors, and a lipophilicity value (LogP) not exceeding 5 [2].

## Results

### Phytochemicals

**Fig 1:**
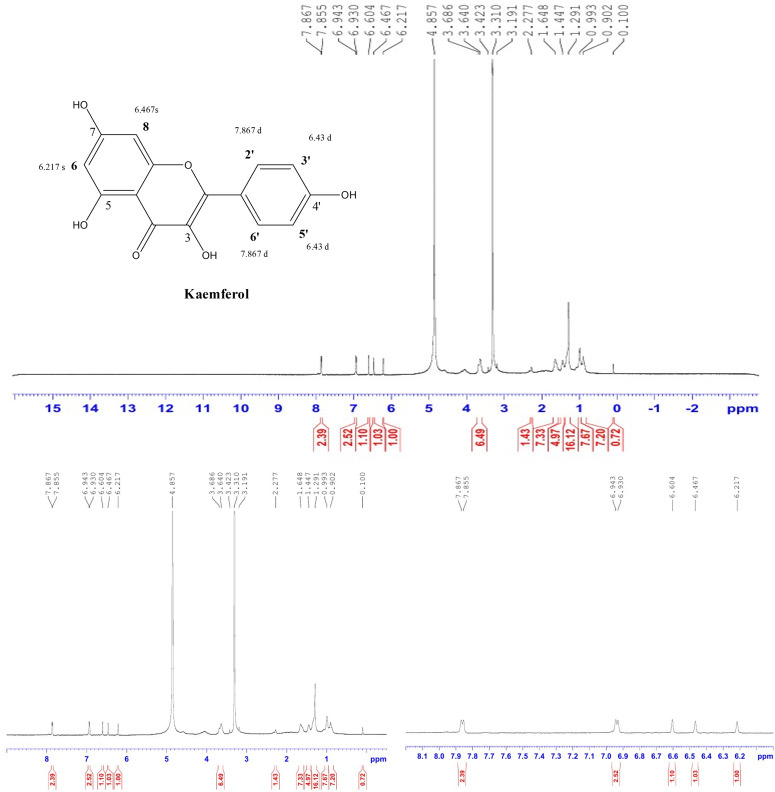
^1^H NMR spectrum of 3,5,7,4’-Tetrahydroxyflavone (Kaempferol) isolated from PSF of methanolic extract of flower of *E. crassipes.*

Compound 1 was a yellowish solid (4.9 mg), Rf = 0.61 (50% Ethyl acetate, 50% Toluene); the obtained ¹H NMR spectra showed consistency with the previously reported data presented in **Table 2**. The ¹H NMR spectrum (CDCl_3_, 600 MHz) showed peak at δ 7.867 (2H, d J = 7.2 Hz, H-2′), δ 6.43 (2H, d J = 7.8 Hz, H-3′), δ 6.467 (1H, s, H-8), and δ 6.217 (1H, s, H-6) (**Fig 1**).

**Table 2:** The ^1^H NMR of the isolated compounds from PSF of methanolic extract of flower of *E. crassipes* compared with reference spectroscopic data.

Compound No	Compound Name	Position	H-NMR Values (δ IN PPM)	Reference Values (δ IN PPM)	Reference
			CDCl_3_, 600 MHz	CD_3_OD, 400 MHz	
1	3,5,7, 4’-Tetrahydroxyflavone (Kaempferol)	H-2′	7.87 (d, 7.2, 2H)	8.05(2H, d, J08.5 Hz)	[35]
		H-3′	6.94 (d, 7.8, 2H)	6.88 (2H, d, J08.5 Hz)	
		H-8	6.47 (s, 1H)	6.35 (1H,s)	
		H-6	6.22 (s, 1H)	6.15(1H,s)	
			**MeOD-d4, 600 MHz**	**CD_3_OD, 500 MHz**	
2	3’,4’,5,7-Tetrahydroxyflavone (Luteolin)	H-6	6.21 (s, 1H)	6.19 (1H, d, J = 2.0 Hz)	[36]
		H-8	6.49 (s, 1H)	6.42 (1H, d, J = 2.0 Hz)	
		H-3	6.55 (s, 1H)	6.52 (1H,s)	
		H-5′	6.90 (d, J = 7.8, 1H)	6.88 (1H, d, J = 8.5 Hz)	
		H-2′, H-6′	7.38 (d, J = 9, 2H)	7.36 (2H, m)	
			**CDCl**_**3**_ **at 600 MHz**	**500 MHz, CDCl_3_**	
3	Stigmasterol	H-3	3.52 (m, 1H)	3.32 (m, 1H)	[37]
		H-6	5.35 (br s, 1H)	5.35 (d, 6.0, 1H)	
		H-18	0.68 (s, 3H)	0.73 (s, 3H)	
		H-19	1.01 (s, 3H)	1.02 (s, 3H)	
		H-22	5.12 (dd, 9, 7.2, 1H)	5.05 (dd, 8.5, 15, 1H)	
		H-23	5.02 (dd, 9, 8.4, 1H)	4.95 (dd, 8.5, 15, 1H)	
		H-26	0.81 (d, 7.2, 3H)	0.83 (d, 3H)	
		H-27	0.79 (d, 7.8, 3H)	0.81 (d, 3H)	
		H-29	0.84 (d, 7.8, 3H)	0.86 (d, 3H)	
			**MeOD-d4, 600 MHz**	**DMSO, 400 MHz**	
4	4- Carboxybenzyl alcohol	H-2, H-6	7.87 (d, 9, 2H)	7.90 (d, 8.2 2H)	[38]
		H-3, H-5	6.81 (d, 8.4, 2H)	7.42 (d, 8.3, 2H)	
			4.58 (s, 2H)	4.57 (s, 2H)	
			**CDCl_3_, 600 MHz**	**DMSO, 400 MHz**	
5	4-methoxybenzaldehyde	H-2, H-6	7.41 (2H, d, J = 7.2 Hz)	7.87 (2H, d, J = 8.6 Hz)	[39]
		H-3, H-5	6.79 (2H, d, J = 7.8 Hz)	7.12 (2H, d, J = 8.6 Hz)	
			3.83 (3H, s)	3.86 (3H, s)	
				9.87 (3H, s)	

**Fig 2:**
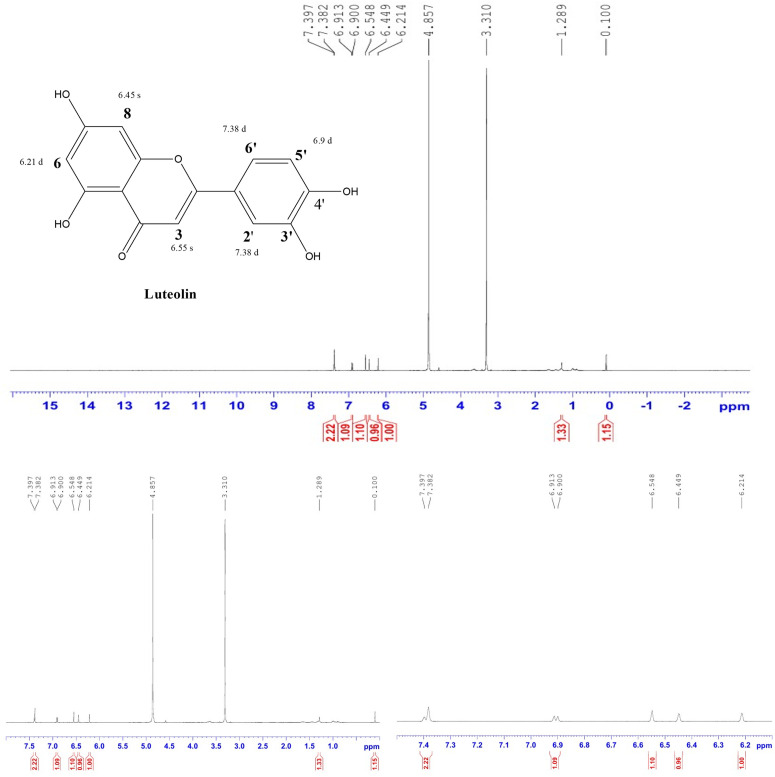
^1^H NMR spectrum of 3’,4’,5,7-Tetrahydroxyflavone (Luteolin) isolated from PSF of methanolic extract of flower of *E. crassipes.*

Compound 2 was a yellowish solid (6.1 mg), Rf = 0.54 (80% Ethyl acetate, 20% Toluene); The spectral profile obtained from ¹H NMR analysis agrees with the previously documented data given in **Table 2**. The ¹H NMR spectrum (MeOD-d4, 600 MHz) showed peak at δ 6.214 (1H, s, H-6), δ 6.499 (1H, s, H-8), δ 6.548 (1H, s, H-3), δ 6.9 (1H, d = 7.8 Hz, H-5′), and δ 7.3825 (2H, d J = 9 Hz, H-2′, H-6′) (**Fig 2**).

**Fig 3:**
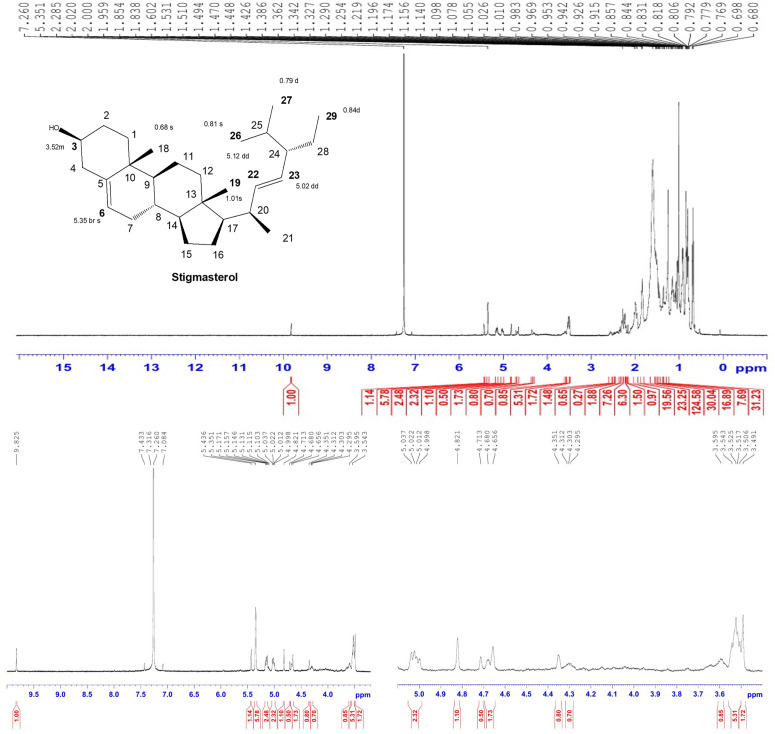
^1^H NMR spectrum of Stigmasterol isolated from PSF of methanolic extract of flower of *E. crassipes.*

Compound 3 was crystalline solid (5.2 mg), Rf = 0.23 (07% Ethyl acetate, 93% Toluene); the observed ¹H NMR resonances aligned with previously published values, as illustrated in **Table 2**. The ¹H NMR spectrum (CDCl_3_, 600 MHz) illustrated peak at δ 3.52 (1H, m, H-3), δ 5.35 (1H, br s, H-6), δ 0.68 (1H, s, H-18), δ 1.01 (1H, s, H-19), δ 5.12 (1H, dd, J = 9 Hz, 7.2 Hz, H-22), δ 5.02 (1H, dd, J = 9 Hz, 8.4 Hz, H-23), δ 0.81 (3H, d, J = 7.2 Hz, H-26), δ 0.79 (3H, d, J = 7.8 Hz, H-27), and δ 0.84 (3H, d, J = 7.8 Hz, H-29) (**Fig 3**).

**Fig 4:**
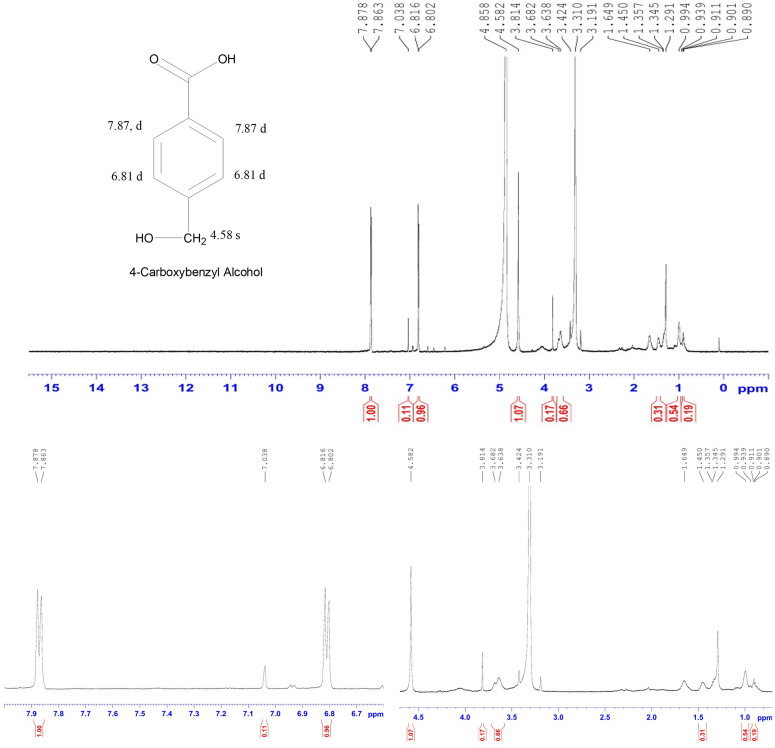
^1^H NMR spectrum of 4-Carboxybenzyl alcohol isolated from PSF of methanolic extract of flower of *E. crassipes.*

Compound 4 was white solid (3.1 mg), Rf = 0.59 (60% Ethyl acetate, 40% Toluene); the ^1^H NMR signals observed for the compound correspond well with the reference data presented in **Table 2**. The ¹H NMR spectrum (MeOD-D4, 600 MHz) showed peak at 7.87 (2H, d J = 9 Hz), 6.81 (2H, d J = 8.4 Hz), and 4.58 (2H, s) (**Fig 4**).

**Fig 5:**
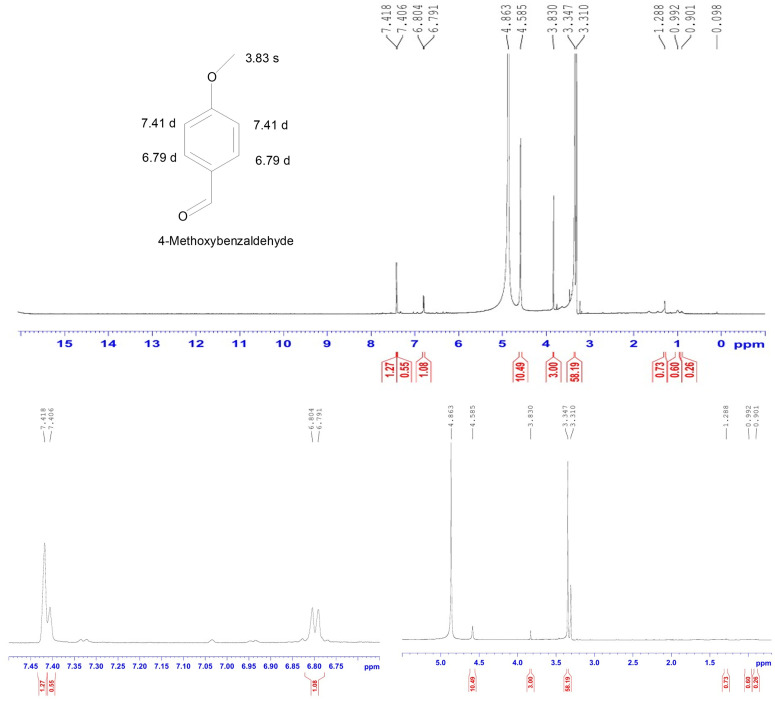
^1^H NMR spectrum of 4-methoxybenzaldehyde isolated from PSF of methanolic extract of flower of *E. crassipes.*

Compound 5 was pale yellow oily liquid (4.7 mg), Rf = 0.54 (50% Ethyl acetate, 50% Toluene); the obtained ^1^H NMR spectral data matched closely with those previously reported and listed in **Table 2**. The ¹H NMR spectrum (MeOD-D4, 600 MHz) showed peak at 7.41 (2H, d, J = 7.2 Hz), 6.79 (2H, d, J = 7.8 Hz), and 3.83 (3H, s) (**Fig 5**).

#### Antimicrobial activities.

**Disc diffusion method (zone of inhibition):** The antimicrobial results revealed that CME displayed broad-spectrum inhibitory activity (**Table 3**). Among Gram-positive organisms, substantial activity was observed against *Bacillus cereus* (10.00 ± 0.82 mm), *Bacillus subtilis* (20.33 ± 1.25 mm), and *Staphylococcus aureus* (7.67 ± 1.25 mm). In Gram-negative organisms, the extract inhibited the growth of *Salmonella paratyphi* (9.00 ± 0.82 mm), *Vibrio parahaemolyticus* (9.67 ± 0.47 mm), and *Shigella dysenteriae* (8.33 ± 0.47 mm). Additionally, antifungal effects were evident against *Candida albicans* (7.00 ± 0.82 mm), *Saccharomyces cerevisiae* (9.00 ± 0.82 mm), and *Aspergillus niger* (11.67 ± 1.70 mm). Although the inhibitory zones were smaller than those produced by the standard drugs ciprofloxacin (for bacteria) and fluconazole (for fungi), the extract’s effects were nonetheless noteworthy.

**Table 3:** Antimicrobial activity of different extracts of *Eichhornia crassipes* flower determined by disc diffusion method (zone of inhibition, mm) and minimum inhibitory concentration (MIC, µL/mL).

Test Microorganisms	Zone of Inhibition (mm)						Minimum Inhibitory Concentration (μL/mL)				
	Standard	CME	PSF	DSF	ESF	ASF	CME	PSF	DSF	ESF	ASF
**Gram Positive Bacteria**											
*Bacillus cereus*	42.33 ± 0.47	10.00 ± 0.82	4.67 ± 0.94	7.33 ± 0.47	8.67 ± 0.94	3.00 ± 0.00	62.5	>500	250	250	>500
*Bacillus megaterium*	32.00 ± 0.82	9.33 ± 0.47	6.67 ± 1.70	8.67 ± 0.47	7.67 ± 0.94	0.00 ± 0.00	125	500	125	250	>500
*Bacillus subtilis*	40.67 ± 0.47	20.33 ± 1.25	5.67 ± 0.94	15.00 ± 0.82	10.00 ± 0.82	4.67 ± 0.47	15.6	500	31.2	125	>500
*Staphylococcus aureus*	42.67 ± 1.25	7.67 ± 1.25	9.67 ± 1.25	17.33 ± 0.47	14.67 ± 1.25	0.00 ± 0.00	250	125	15.6	62.5	>500
*Sarcina lutea*	40.00 ± 0.82	11.00 ± 1.63	8.33 ± 0.47	11.00 ± 0.82	11.00 ± 1.63	0.00 ± 0.00	62.5	125	62.5	62.5	>500
**Gram Negative Bacteria**											
*Salmonella paratyphi*	43.00 ± 0.82	9.00 ± 0.82	8.00 ± 0.82	8.67 ± 0.47	8.67 ± 1.70	2.33 ± 0.47	125	250	125	125	>500
*Salmonella typhi*	42.67 ± 0.94	8.33 ± 0.47	12.67 ± 1.25	9.00 ± 0.82	4.33 ± 0.47	0.00 ± 0.00	125	62.5	125	>500	>500
*Vibrio parahaemolyticus*	41.33 ± 0.47	9.67 ± 0.47	4.67 ± 0.94	8.33 ± 0.94	8.67 ± 0.58	2.67 ± 0.47	125	>500	125	125	>500
*Escherichia coli*	40.33 ± 1.25	12.67 ± 1.25	8.33 ± 0.94	6.67 ± 1.15	6.67 ± 0.94	0.00 ± 0.00	62.5	250	500	500	>500
*Vibrio mimicus*	37.00 ± 1.63	11.33 ± 0.47	10.33 ± 1.25	7.33 ± 0.47	6.00 ± 0.00	0.00 ± 0.00	62.5	125	250	500	>500
*Shigella dysenteriae*	38.00 ± 0.82	8.33 ± 0.47	10.67 ± 1.25	9.67 ± 0.94	7.33 ± 1.53	5.00 ± 0.82	125	62.5	125	250	500
*Pseudomonas aeruginosa*	39.00 ± 2.16	11.67 ± 0.47	8.67 ± 0.47	8.00 ± 0.82	8.00 ± 0.82	0.00 ± 0.00	62.5	125	250	250	>500
*Shigella boydii*	40.67 ± 0.47	11.67 ± 0.94	9.33 ± 1.25	9.33 ± 0.47	11.67 ± 0.47	0.00 ± 0.00	62.5	125	125	125	>500
**Fungi**											
*Saccharomyces cerevisiae*	36.33 ± 1.25	9.00 ± 0.82	7.67 ± 0.47	8.00 ± 0.82	6.67 ± 0.47	6.33 ± 0.47	125	250	250	500	500
*Candida albicans*	40.00 ± 0.82	7.00 ± 0.82	6.67 ± 0.47	3.67 ± 0.47	7.67 ± 1.25	4.00 ± 0.82	250	500	>500	250	>500
*Aspergillus niger*	48.00 ± 2.16	11.67 ± 1.70	4.00 ± 0.82	7.67 ± 0.47	11.00 ± 0.82	5.67 ± 0.47	62.5	>500	250	62.5	>500

Zone of Inhibition results are expressed as Mean ± SD. For Gram positive and negative bacteria Ciprofloxacin used as standard, and for fungi Fluconazole used as standard.

**Minimum inhibitory concentration (MIC) assay:** Among the Gram-positive bacteria, the DSF exhibited the most potent activity, particularly against *B. subtilis* and *S. aureus*, with the lowest MIC value of 15.6 µL/mL. The CME also demonstrated notable activity against *B. subtilis* (15.6 µL/mL) and *S. lutea* (62.5 µL/mL). In contrast, the ASF showed no significant inhibition against Gram-positive bacteria, with MIC values exceeding 500 µL/mL in all cases.

For Gram-negative bacteria, moderate antimicrobial activity was observed across CME, PSF, and DSF fractions. The lowest MIC values (62.5 µL/mL) were recorded for CME against *E. coli*, *V. mimicus*, and *P. aeruginosa*. The PSF exhibited selective activity, with an MIC of 62.5 µL/mL against *S. typhi* and *S. dysenteriae*. However, higher MIC values (≥250 µL/mL) were generally observed for ESF and ASF, indicating comparatively weaker activity of these fractions against Gram-negative organisms.

In antifungal assays, the CME fraction showed moderate activity against *A. niger* (62.5 µL/mL), whereas other fractions demonstrated limited efficacy. The DSF fraction exhibited moderate inhibition against *S. cerevisiae* (250 µL/mL), while most other combinations, particularly ASF, showed MIC values of ≥500 µL/mL, suggesting minimal antifungal potential.

Overall, the results indicate that the DSF and CME fractions possess comparatively stronger antimicrobial activity, while PSF and ESF exhibit moderate, organism-specific effects, and ASF remains largely inactive. The observed variability in MIC values highlights the influence of solvent polarity and phytochemical composition on antimicrobial efficacy.

### Thrombolytic activities

**Fig 6:**
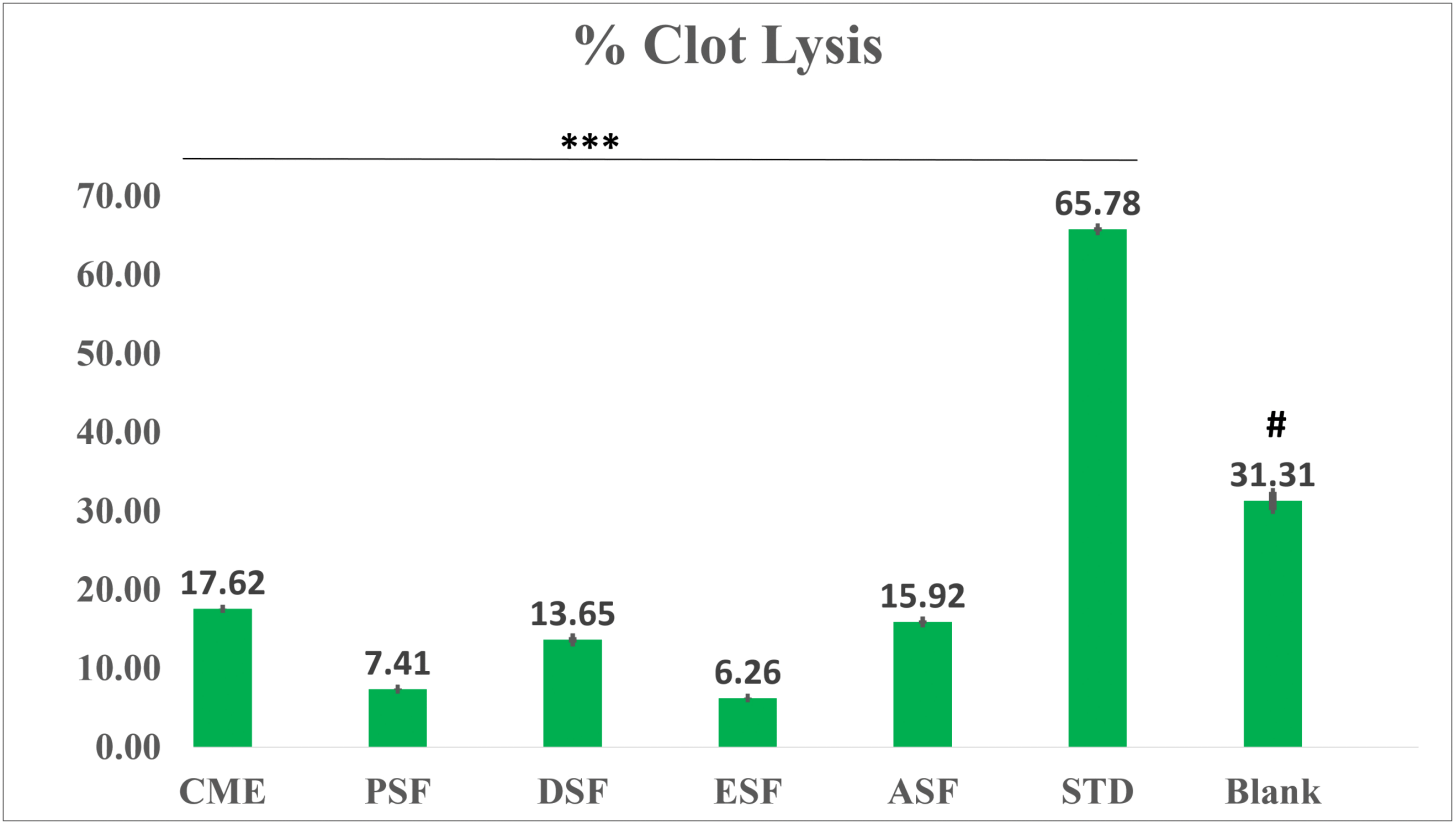
Thrombolytic activities of different fraction of methanolic extract of flower of *E. crassipes.*

The thrombolytic activity of *E. crassipes* flower extracts and their fractions was evaluated *in vitro* and expressed as percentage of clot lysis (**Fig 6**). The blank control produced 31.28% clot lysis, whereas the reference drug SK showed 66.67% clot lysis, confirming the validity of the assay. Among the tested samples, CME exhibited 17.68% clot lysis, followed by ASF (15.80%), DSF (13.32%), PSF (7.40%), and ESF (6.23%). Overall, the extracts and fractions demonstrated measurable but moderate thrombolytic activity compared with the standard SK. These findings suggest that the thrombolytic constituents of *E. crassipes* flowers are more concentrated in the ethyl acetate fraction.

### Antidiarrheal activities

The antidiarrheal activity of CME extract was evaluated at three doses (200, 400, and 600 mg/kg) compared to a standard drug and control group over a 4-hour period (**Table 4**). The control group showed a progressive increase in diarrheal count from 1.00 ± 0.41 at 1 hour to 4.50 ± 0.50 at 4 hours. CME 200 mg/kg demonstrated moderate reduction in diarrheal counts, with 50% reduction at 1 hour but no effect at 3 hours. CME 400 mg/kg showed stronger activity, with 75% reduction at 1 hour and statistically significant 77.78% reduction at 2 hours (p < 0.05). The highest dose of CME 600 mg/kg produced the most pronounced effects, completely preventing diarrhea at 1 hour (100% reduction) and maintaining significant reductions of 77.78% at 2 hours (p < 0.05), 55.56% at 3 hours, and 38.89% at 4 hours. The standard drug exhibited comparable efficacy to CME 600 mg/kg, showing 100% reduction at 1 hour and maintaining statistically significant 61.11% reduction at 4 hours (p < 0.05).

**Table 4:** Anti-diarrheal activites of methanolic crude extract of flower of *E. crassipes* through Castor oil induced diarrheal assay.

Groups	Average diarrheal count				% Reduction in diarrheal count			
	1 h	2 h	3 h	4 h	1 h	2 h	3 h	4 h
Control	1.00 + 0.41	2.25 + 0.48	2.25 + 0.48	4.50 + 0.50	–	–	–	–
CME 200	0.50 + 0.50	1.75 + 0.85	2.25 + 1.11	4.00 + 0.82	50.00	22.22	0.00	11.11
CME 400	0.25 + 0.25	0.50 + 0.50*	2.00 + 0.41	3.75 + 0.48	75.00	77.78	11.11	16.67
CME 600	0.00	0.50 + 0.50*	1.00 + 1.00	2.75 + 1.18	100.00	77.78	55.56	38.89
STD	0.00	0.75 + 0.25*	1.25 + 0.48	1.75 + 0.63*	100.00	66.67	44.44	61.11

Results express as (Average ± SEM) while N = 4 and levels of significance were denoted as *p < 0.05, **p < 0.01, and ***p < 0.001.

### Molecular-docking

**Fig 7:**
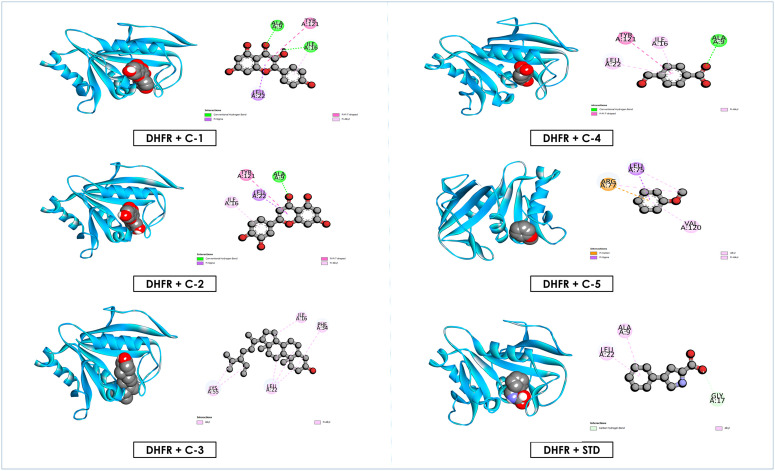
Molecular docking interactions of the isolated compounds with dihydrofolate reductase (DHFR). The figure illustrates both 3D binding conformations within the active site pocket and corresponding 2D interaction maps, highlighting key interactions such as hydrogen bonding, hydrophobic contacts, and π–alkyl interactions between the ligands and amino acid residues located in the DHFR catalytic region.

**Fig 8:**
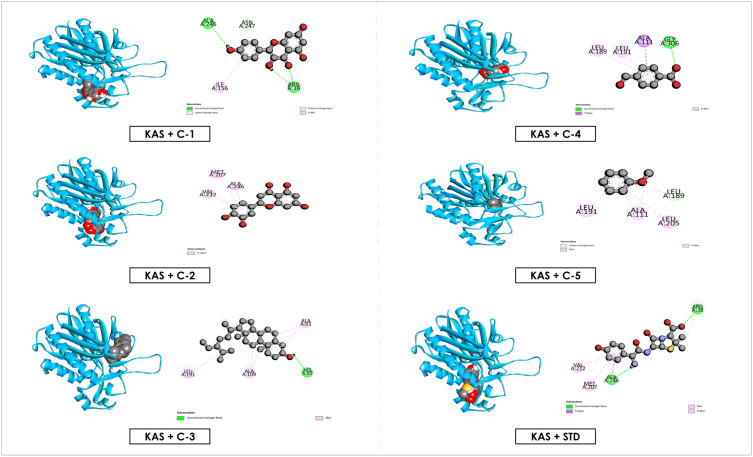
Docking interactions of the isolated compounds with β-ketoacyl-ACP synthase (KAS). The 3D docking poses and 2D interaction diagrams show the orientation of the ligands within the KAS active site and indicate important hydrogen bonds and hydrophobic interactions with catalytic residues, suggesting potential binding stability.

**Fig 9:**
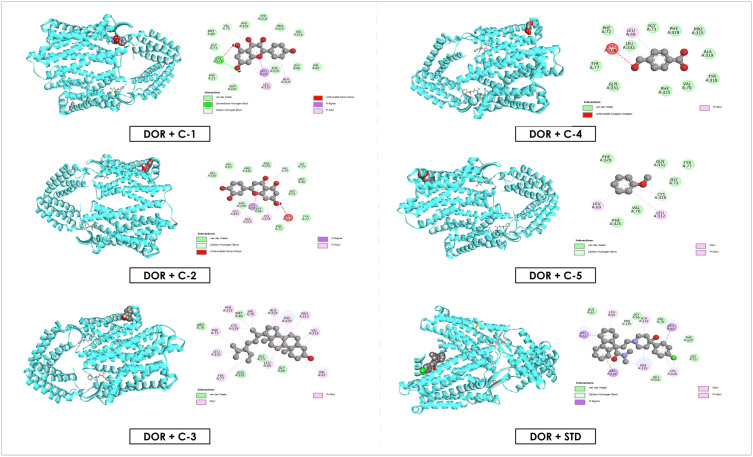
Docking interactions of the isolated compounds with the delta-opioid receptor (DOR). The figure presents three-dimensional ligand binding orientations along with two-dimensional interaction maps, illustrating hydrogen bonding, hydrophobic contacts, and other stabilizing interactions between the ligands and residues within the receptor binding pocket.

**Fig 10:**
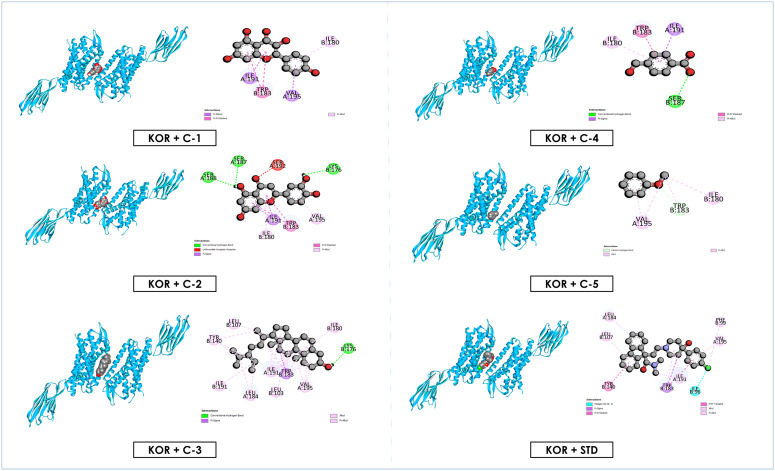
Docking interactions of the isolated compounds with the kappa-opioid receptor (KOR). The 3D binding poses and corresponding 2D interaction diagrams demonstrate the positioning of the ligands within the receptor binding cavity and highlight key amino acid interactions contributing to ligand stabilization, including hydrogen bonding and hydrophobic contacts.

**Fig 11:**
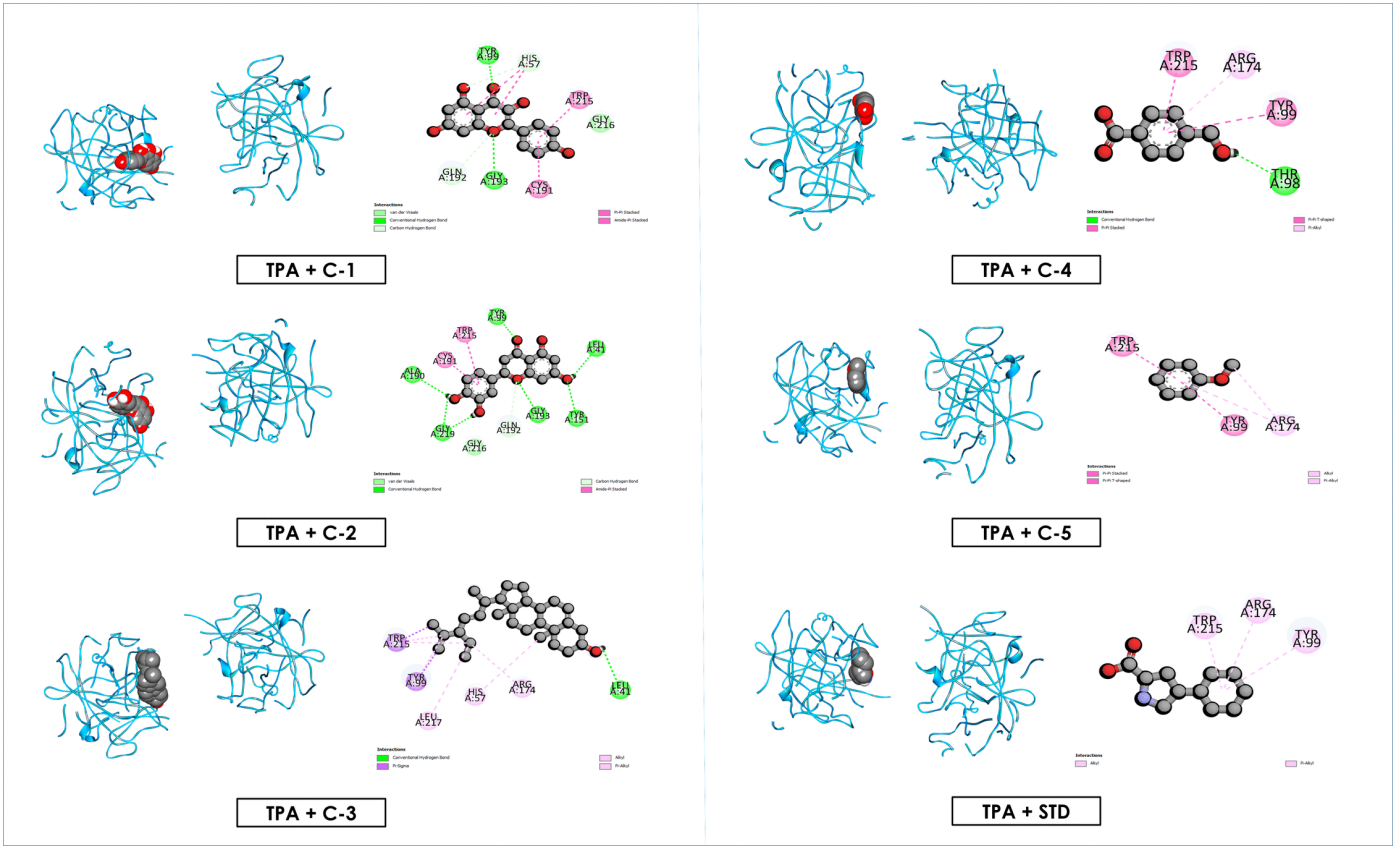
Docking interactions of the isolated compounds with tissue plasminogen activator (TPA). The 3D ligand–receptor complexes and 2D interaction maps depict the binding orientation of the compounds within the TPA active site and illustrate hydrogen bonding and hydrophobic interactions with surrounding residues, which may contribute to the predicted binding affinity.

Molecular docking investigations of the isolated constituents from the PSF of *Eichhornia crassipes* flower demonstrated diverse binding behaviors toward antimicrobial, antidiarrheal, and thrombolytic protein targets (**Table 5**, **Figs 7**–**11**). Stigmasterol emerged as the most active compound, with docking scores of –8.4 (DHFR), –7.5 (KAS), –8.4 (delta-opioid receptor), –10.3 (kappa-opioid receptor), and –7.7 kcal/mol (tPA). Luteolin also displayed strong and consistent affinities across all tested proteins, ranging between –7.5 and –9.1, while Kaempferol recorded moderately high interactions spanning –6.8 to –8.4. In contrast, the simpler phenolic molecules showed weaker binding tendencies; 4-carboxybenzyl alcohol yielded values between –5.4 and –6.5, and 4-methoxy benzene demonstrated the lowest scores (–4.3 to –5.2).

**Table 5:** Molecular docking of isolated compounds from PSF of methanolic extract of flower of *E. crassipes* against different target receptors.

SL.NO	Compound Name	Pub chem ID	Antimicrobial		Antidiarrheal		Thrombolytic
			Dihydrofolate Reductase (DHFR) [PDB ID4M6J]	Beta-ketoacyl-ACP synthases (KAS) [PDB ID:1HNJ]	Human Delta-Opioid Receptor (DOR) [PDB ID: 4RWD]	Kappa Opioid Receptor (KOR) [PDB ID: 6VI4]	Tissue Plasminogen Activator (TPA) [PDB ID: 1A5H]
1	Kaempferol	5280863	−7.8	−6.8	−8.4	−8.4	−7.6
2	Luteolin	5280445	−8.1	−7.5	−8.1	−9.1	−8
3	Stigmasterol	5280794	−8.4	−7.5	−8.4	−10.3	−7.7
4	4-Carboxybenzyl alcohol	7009454	−5.4	−5.6	−6.5	−5.7	−6.1
5	4-Methoxy benzene	7519	−4.3	−4.6	−5.2	−4.9	−5.2
STD	Ciprofloxacin	2764	−8.2				
	Amoxicillin	33613		−7.1			
	Loperamide	3955			−8.5	−9.1	
	Streptokinase	482240142					−5.8

The performance of the phytochemicals was comparable with standard reference ligands. Ciprofloxacin and amoxicillin interacted strongly with antimicrobial proteins (–8.2 and –7.1, respectively), loperamide showed excellent binding to opioid receptors (–8.5 and –9.1), while streptokinase exhibited a lower affinity (–5.8) with tPA compared to Stigmasterol and Luteolin. These results indicate that the sterols and flavonoids from *E. crassipes* may interact with the selected targets with comparable predicted affinities; however, these results represent computational estimates and require experimental validation.

## Discussion

The ^1^H NMR spectrum (CDCl3, 600 MHz) of **Compound 1** provides clear and distinct signals that help confirm its flavonoid structure. The doublet at 7.867 ppm (J = 7.2 Hz) corresponds to the two protons at C2′ on the B-ring, indicating that these protons are adjacent to each other in an ortho relationship. Similarly, the doublet at 6.43 ppm (J = 7.8 Hz) is assigned to the two protons at C3′ on the B-ring, which are also in an ortho relationship, as indicated by the similar coupling constant. These signals suggest that the B-ring of **5,7,4’-Tetrahydroxyflavone** is symmetrically substituted with protons at positions 2′ and 3′. The singlet at 6.467 ppm corresponds to the proton at C8 on the A-ring, which is isolated and does not couple with adjacent protons, as expected for a proton in this position. Another singlet at 6.217 ppm is assigned to the proton at C6, which is also isolated and does not show coupling with nearby protons. This further supports the typical pattern for a flavonoid structure, where protons on the A-ring and B-ring exhibit distinct coupling behaviors. The overall pattern of signals, with doublets for the protons on the B-ring and singlets for those on the A-ring, is consistent with the known proton environments of **3,5,7,4’-Tetrahydroxyflavone** or **Kaempferol**. This confirms that Kaempferol possesses a hydroxylated B-ring and a conjugated A-ring, as well as a typical flavonoid structure.

The ^1^H NMR spectrum (MeOD-d_4_, 600 MHz) of **Compound 2** reveals several characteristic signals that confirm its flavonoid structure. The singlet at 6.214 ppm corresponds to the aromatic proton H-6, located on the A-ring of the flavonoid. The absence of coupling suggests that this proton has no neighboring protons, consistent with the substitution pattern of the ring. The singlet at 6.499 ppm is assigned to H-8, another proton on the A-ring, indicating a similar isolated environment. The difference in chemical shifts between H-6 and H-8 is influenced by the electron density distribution within the ring system. The singlet at 6.548 ppm is attributed to H-3, positioned within the central ring system of the flavonoid. This proton is strongly influenced by the extended conjugation of the structure, which leads to its characteristic downfield shift. The lack of splitting further supports its placement, as it is not adjacent to another proton. The doublet at 6.9 ppm (J = 7.8 Hz) corresponds to H-5′, located on the B-ring of the flavonoid. The coupling constant of 7.8 Hz suggests an ortho relationship between H-5′ and an adjacent proton, reinforcing the expected substitution pattern of the B-ring. The doublet at 7.3825 ppm (J = 9 Hz) is assigned to H-2′ and H-6′, which are also in an ortho arrangement on the B-ring. The coupling constant of 9 Hz supports their close positioning, confirming the expected connectivity of the ring system. Together, these signals confirm the structure of **3’,4’,5,7-Tetrahydroxyflavone** or **Luteolin**, a hydroxylated flavonoid with a defined arrangement of protons on both the A-ring and B-ring. The pattern of singlets and doublets aligns with the expected electronic environment and substitution pattern, reinforcing the molecular framework of **Luteolin**.

The ^1^H NMR spectrum (CDCl_3_, 600 MHz) of **Compound 3** exhibits characteristic signals confirming its steroidal framework, hydroxyl functionality, and unsaturated side chain. A multiplet at δ 3.517 ppm is assigned to H-3, indicating the presence of a hydroxyl (-OH) group at C-3, a typical feature of sterols. The broad singlet at δ 5.351 ppm corresponds to H-6, confirming a double bond between C-5 and C-6 in the steroid nucleus. The singlets at δ 0.680 and δ 1.010 ppm correspond to H-18 and H-19, which are characteristic methyl groups in the sterol core. The side-chain double bond is confirmed by double doublets at δ 5.124 (J = 9, 7.2 Hz) and δ 5.017 (J = 9, 8.4 Hz), attributed to H-22 and H-23, indicating a double bond between C-22 and C-23. The aliphatic side-chain methyl groups appear as doublets at δ 0.812 (J = 7.2 Hz, H-26), δ 0.786 (J = 7.8 Hz, H-27), and δ 0.838 (J = 7.8 Hz, H-29), confirming the presence of an isopropyl terminal at the end of the side chain. The observed chemical shifts, coupling constants, and splitting patterns are consistent with the structure of **Stigmasterol**, confirming its hydroxylated sterol core, double bonds at C-5, C-6, C-22, and C-23, and a branched aliphatic side chain.

The ^1^H-NMR spectrum of **Compound 4**, isolated from the dichloromethane fraction of *Eichhornia crassipes* flower, was recorded in MeOD-d4 at 600 MHz. The spectrum displayed a well-resolved pattern characteristic of a para-substituted benzene ring. Two doublets were observed at δ 7.87 ppm (d, J = 9 Hz, 2H) and δ 6.81 ppm (d, J = 8.4 Hz, 2H), corresponding to the aromatic protons at positions H-2/H-6 and H-3/H-5, respectively. The coupling constants (~9 Hz) confirm ortho relationships, while the symmetry of the two equivalent sets of protons is consistent with para substitution on the aromatic ring. In addition, a singlet resonance at δ 4.58 ppm integrating for two protons was assigned to the benzylic methylene group (–CH2OH). The absence of splitting in this signal indicates that the two protons are magnetically equivalent, while the deshielding effect of the adjacent aromatic carboxyl group accounts for its chemical shift in the downfield region. Overall, the splitting pattern and chemical shifts are fully consistent with the proposed structure of **4-Carboxybenzyl alcohol**, confirming the presence of a para-substituted benzyl system with both carboxyl and hydroxymethyl functional groups in expected positions.

The 1H NMR spectrum (MeOD-d4, 600 MHz) of **Compound 5** exhibits signals that clearly reflect its symmetrical para-substituted aromatic structure. The doublet at 7.41 ppm (2H, J = 7.2 Hz) is attributed to the aromatic protons located ortho to the methoxy group (positions 2 and 6), while the second doublet at 6.79 ppm (2H, J = 7.8 Hz) corresponds to the protons at positions 3 and 5. These splitting patterns arise due to typical ortho-coupling interactions between adjacent aromatic protons, and the near-equal coupling constants further support the symmetrical nature of the molecule. Additionally, a singlet observed at 3.83 ppm integrating for three protons corresponds to the methoxy group (-OCH3) attached to the para position of the benzene ring. The absence of coupling confirms that the methoxy protons are isolated from other hydrogen interactions. The overall pattern—two doublets for the aromatic protons and a singlet for the methoxy group—is characteristic of a para-substituted aromatic ether and supports the identity of the compound as **4-methoxybenzaldehyde**. The symmetry simplifies the spectrum, resulting in clearly resolved and easily assignable peaks.

Again the present study establishes the broad pharmacological potential of *Eichhornia crassipes* flower extract (CME), demonstrating significant antidiarrheal, antimicrobial, and thrombolytic activities. The marked dose-dependent suppression of castor oil–induced diarrhea indicates that CME influences intestinal motility and fluid secretion. Phytoconstituents such as flavonoids, tannins, alkaloids, and phenolics are well known for their ability to inhibit prostaglandin synthesis, enhance water and electrolyte reabsorption, and reduce gastrointestinal hyperactivity [40]. Such mechanisms are consistent with the profound effect observed at higher doses, which were comparable to the standard loperamide.

The antimicrobial investigation revealed that the extracts exhibited moderate to significant zones of inhibition (ZOI), which were largely supported by corresponding MIC values, indicating genuine antimicrobial potential. Among the fractions, DSF and CME demonstrated superior activity, evidenced by larger inhibition zones (up to ~20 mm) and lower MIC values (as low as 15.6 µL/mL), particularly against Gram-positive bacteria such as *B. subtilis* and *S. aureus*. This trend aligns with previous studies reporting that moderately non-polar fractions often concentrate bioactive phytoconstituents with strong antimicrobial effects [41,42]. The PSF fraction showed selective moderate activity, with notable effects against *S. typhi* and *S. dysenteriae*, while remaining less active against other strains. This organism-specific behavior may be attributed to the presence of lipophilic compounds, such as terpenoids and fatty acid derivatives, which can disrupt microbial cell membranes but may have limited diffusion capacity in agar media [43]. Consequently, some discrepancies between ZOI and MIC values were observed, which is common in plant extract studies due to differences in compound diffusion, solubility, and interaction with agar matrices [44].

In general, Gram-positive bacteria were more susceptible than Gram-negative bacteria, as reflected by larger ZOI and lower MIC values. This can be explained by the structural differences in their cell envelopes; Gram-negative bacteria possess an outer membrane rich in lipopolysaccharides that restricts the penetration of many phytochemicals [45]. Additionally, the absence of this outer barrier in Gram-positive bacteria facilitates enhanced interaction of lipophilic and phenolic compounds with the cytoplasmic membrane, leading to increased permeability and cellular leakage [41,46]. The comparatively weaker antifungal activity observed may be due to the rigid chitin-containing cell wall of fungi, which reduces susceptibility to plant-derived compounds [47]. The antimicrobial mechanisms of the active fractions are likely multifactorial, involving cell membrane disruption, protein denaturation, enzyme inhibition, and interference with nucleic acid synthesis, depending on the phytochemical composition. Compounds such as flavonoids, phenolics, and sterols—previously identified in similar plant extracts—are known to exert antimicrobial effects through membrane permeability alteration and oxidative stress induction [48,49].

The antimicrobial effects of the PSF can be further rationalized by the presence of identified compounds such as kaempferol, luteolin, and stigmasterol. Flavonoids like kaempferol and luteolin are reported to exert antimicrobial activity through multiple mechanisms, including inhibition of nucleic acid synthesis, disruption of energy metabolism, and induction of oxidative stress [50,51]. Meanwhile, sterol compounds such as stigmasterol may contribute to membrane destabilization due to their lipophilic nature, potentially altering membrane fluidity and integrity. These findings suggest a multifactorial and potentially synergistic mode of action within the crude fraction. Overall, the findings suggest that solvent polarity plays a critical role in extracting bioactive compounds, with DSF and CME being the most effective fractions. The observed variation between ZOI and MIC further emphasizes the importance of combining diffusion-based and dilution-based assays to accurately evaluate antimicrobial potential.

The thrombolytic assay revealed that the extracts of *E. crassipes* flowers possess measurable clot-dissolving activity *in vitro*. Although the observed activity was lower than that of the standard SK, the results indicate the presence of phytochemicals capable of influencing fibrinolytic processes. Previous studies have suggested that polyphenolic compounds, particularly flavonoids and tannins, may contribute to thrombolytic effects through mechanisms such as plasminogen activation, fibrin degradation, or inhibition of platelet aggregation [52]. The presence of such phytochemicals in *E. crassipes* flowers may therefore account for the clot lysis activity observed in this study. Although certain fractions exhibited comparatively higher thrombolytic activity, the PSF fraction was selected for phytochemical investigation because it yielded isolable compounds in sufficient quantities for structural characterization and subsequent molecular docking and ADMET analysis. This fraction enabled the identification of stigmasterol, kaempferol, and luteolin, which were further evaluated to explore their possible interactions with relevant biological targets. These findings provide preliminary insight into the potential role of these phytochemicals in the observed biological activity and support further investigation of *E. crassipes* flowers as a source of pharmacologically relevant compounds.

The docking outcomes suggest that Stigmasterol, Luteolin, and Kaempferol may contribute to the pharmacological activities observed in the PSF of *E. crassipes*. Stigmasterol, in particular, showed the strongest predicted binding affinity for the kappa opioid receptor (–10.3 kcal/mol), indicating a possible interaction with opioid-mediated pathways involved in intestinal motility and thereby providing a hypothetical mechanistic explanation for the antidiarrheal activity observed in experimental assays. Luteolin also displayed notable predicted interactions with both delta- and kappa-opioid receptors (–8.1 and –9.1 kcal/mol), which is consistent with previous reports suggesting that certain flavonoids may influence opioid and prostaglandin signaling pathways involved in gastrointestinal regulation (**Table 5**, **Figs 9 and 10**) [53].

The antimicrobial docking profiles of these flavonoids (Luteolin and Kaempferol) and sterols (Stigmasterol) indicated moderate predicted binding interactions (–7.5 to –8.4 kcal/mol) with antimicrobial targets. While these findings provide supportive computational evidence, they should not be interpreted as definitive proof of bacteriostatic activity, and experimental validation through enzyme inhibition or mechanistic assays would be required. Nevertheless, these predicted interactions align with previous reports describing how polyphenolic and sterol-based metabolites may disrupt bacterial membranes or interfere with folate metabolism and nucleic acid synthesis (**Table 5**, **Figs 7 and 8**) [41,54].

Similarly, docking against the thrombolytic target suggested that Stigmasterol, Luteolin, and Kaempferol may interact with the tissue plasminogen activator (TPA) binding site, with predicted affinities ranging from –7.6 to –8.0 kcal/mol (**Table 5**, **Fig 11**). Although these values were higher than the calculated score for streptokinase in this docking model, such comparisons should be interpreted cautiously because computational docking scores do not necessarily translate directly into biological potency. These findings therefore provide preliminary hypotheses that may help explain the moderate *in vitro* clot-lysis activity observed and warrant further biochemical investigation [23]. It is important to note that docking scores reflect binding propensity rather than pharmacological efficacy and should therefore be interpreted as hypothesis-generating rather than confirmatory evidence.

The pharmacokinetic characteristics of the isolated phytochemicals were further explored through in silico ADMET analysis, which provides preliminary insights into the absorption, distribution, metabolism, excretion, and toxicity profiles of candidate molecules during early drug discovery. The predicted absorption parameters indicated generally favorable oral bioavailability for most compounds. Kaempferol and Luteolin demonstrated moderate aqueous solubility and good predicted intestinal absorption (>74–81%), whereas Stigmasterol showed lower solubility due to its highly lipophilic steroidal structure (**Table 6**). This elevated lipophilicity (high LogP) is known to reduce aqueous solubility and may adversely affect oral bioavailability despite favorable membrane permeability, a common limitation observed in sterol-type compounds [55,56]. Such reduced solubility is a common limitation of sterol-type molecules and may influence their oral bioavailability despite good membrane permeability. The Caco-2 permeability predictions suggested that several of the investigated compounds are capable of crossing intestinal epithelial barriers, while the predicted P-glycoprotein interactions indicate that flavonoids such as Kaempferol and Luteolin may be subjected to efflux transport mechanisms that could influence their intracellular concentration. Similar transport interactions have been widely reported for polyphenolic compounds during pharmacokinetic evaluation [57,58].

**Table 6:** ADME/T of isolated compounds from PSF of methanolic extract of flower of *E. crassipes.*

Properties	Model name (Unit)	Kaempferol	Luteolin	Stigmasterol	4-Carboxybenzyl alcohol	4-Methoxy benzene
**Absorption**	Water solubility (log mol/L)	−3.04	−3.094	−6.682	−1.931	−1.608
	Caco2 permeability (log Papp in 10−6 cm/s)	0.032	0.096	1.213	1.092	1.529
	Intestinal absorption (human) (% Absorbed)	74.29	81.13	94.97	82.432	96.278
	Skin Permeability (log Kp)	−2.735	−2.735	−2.783	−2.734	−1.814
	P-glycoprotein substrate	Yes	Yes	No	No	No
	P-glycoprotein I inhibitor	No	No	Yes	No	No
	P-glycoprotein II inhibitor	No	No	Yes	No	No
**Distribution**	VDss (human) (log L/kg)	1.274	1.153	0.178	−1.572	0.106
	Fraction unbound (human) (Fu)	0.178	0.168	0	0.67	0.387
	BBB permeability	−0.939	−0.907	0.771	−0.316	0.47
	CNS permeability	−2.228	−2.251	−1.652	−2.871	−1.791
**Metabolism**	CYP2D6 substrate	No	No	No	No	No
	CYP3A4 substrate	No	No	Yes	No	No
	CYP1A2 inhibitior	Yes	Yes	No	No	No
	CYP2C19 inhibitior	No	No	No	No	No
	CYP2C9 inhibitior	No	Yes	No	No	No
	CYP2D6 inhibitior	No	No	No	No	No
	CYP3A4 inhibitor	No	No	No	No	No
**Excretion**	Total Clearance (log mL/min/kg)	0.477	0.495	0.618	0.276	0.271
	Renal OCT2 substrate	No	No	No	No	No
**Toxicity**	AMES toxicity	No	No	No	No	No
	Max. tolerated dose (human) (log mg/kg/day)	0.531	0.499	−0.664	1.534	1.173
	hERG I inhibitor	No	No	No	No	No
	hERG II inhibitor	No	No	Yes	No	No
	Oral Rat Acute Toxicity (LD50) (mol/kg)	2.449	2.455	2.54	1.871	1.915
	Oral Rat Chronic Toxicity (LOAEL) (log mg/kg_bw/day)	2.505	2.409	0.872	2.745	1.964
	Hepatotoxicity	No	No	No	No	No
	Skin Sensitisation	No	No	No	No	Yes
	T.Pyriformis toxicity (log ug/L)	0.312	0.326	0.433	0.231	−0.119
	Minnow toxicity (log mM)	2.885	3.169	−1.675	2.548	1.51
	Molecular weight (g/mol)	286.24	286.24	412.69	151.14	108.14
**Drug-likeness**	Num. H-bond acceptors	6	6	1	3	1
	Num. H-bond donors	4	4	1	1	0
	topological polar surface area (TPSA) (Å²)	111.13	111.13	20.23	60.36	9.23
	Consensus Log Po/w	1.58	1.73	6.98	0.74	1.88
	Lipinski’s RO5 violation	Yes; 0 violation	Yes; 0 violation	Yes; 1 violation: MLOGP>4.15	Yes; 0 violation	Yes; 0 violation
	Bioavailability Score (%)	0.55	0.55	0.55	0.85	0.55

Distribution and metabolic predictions provided additional insights into the systemic behavior of the identified phytochemicals. Kaempferol and Luteolin displayed moderate predicted volumes of distribution and limited blood–brain barrier permeability, suggesting that their pharmacological effects are more likely to occur through systemic circulation rather than central nervous system interactions. Conversely, Stigmasterol showed higher predicted lipophilicity and potential blood–brain barrier penetration, which may influence tissue distribution patterns. Cytochrome P450 metabolism predictions indicated that most of the compounds are not substrates of CYP2D6, while Stigmasterol may undergo metabolism through CYP3A4, the major enzyme involved in xenobiotic metabolism [59]. Toxicity prediction models suggested generally favorable safety profiles, with no predicted AMES mutagenicity or hepatotoxicity for the evaluated molecules, although a possible hERG II inhibition signal for Stigmasterol indicates that further experimental safety evaluation would be necessary [56]. Drug-likeness analysis based on Lipinski’s rule of five demonstrated that Kaempferol and Luteolin satisfy the criteria commonly associated with orally active compounds, whereas Stigmasterol showed a single violation due to high lipophilicity, a characteristic frequently observed among steroid-like natural products [57]. Such high lipophilicity may also result in poor dissolution rate and variable absorption, necessitating formulation strategies to enhance its pharmacokinetic performance [56].

**Fig 12:**
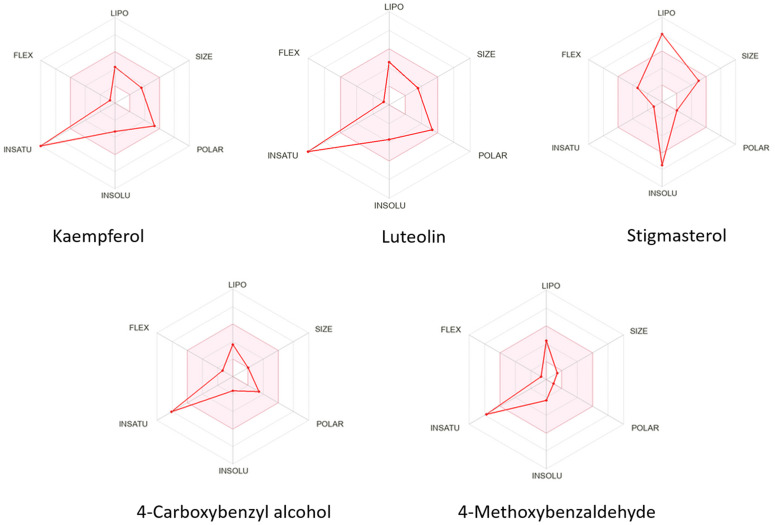
Radar images of isolated from PSF of methanolic extract of flower of *E. crassipes.*

**Fig 13:**
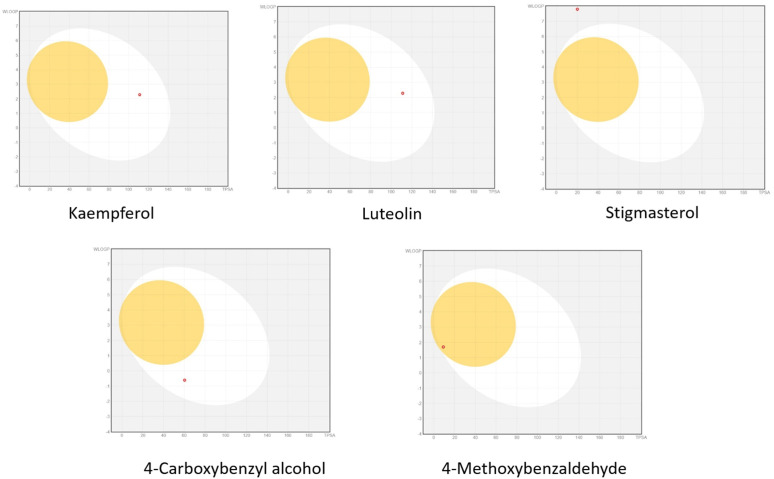
Boiled egg images of isolated from PSF of methanolic extract of flower of *E. crassipes.*

The bioavailability radar plot (**Fig 12**) further illustrated the physicochemical drug-likeness of the identified compounds by integrating key parameters such as lipophilicity, polarity, molecular size, solubility, flexibility, and saturation. Kaempferol and Luteolin were largely positioned within the optimal physicochemical region predicted by the radar model, indicating balanced drug-like properties [60]. In contrast, Stigmasterol showed deviations primarily related to excessive lipophilicity and reduced polarity, which may contribute to its limited aqueous solubility. Complementary evaluation using the BOILED-Egg model (**Fig 13**) provided additional insight into gastrointestinal absorption and blood–brain barrier permeability. In this model, Kaempferol and Luteolin were predicted to lie within the white region associated with a high probability of passive gastrointestinal absorption while remaining outside the yellow “yolk” region representing brain penetration. This pattern suggests that these flavonoids may exert pharmacological effects primarily through systemic exposure rather than central nervous system activity. Collectively, the ADMET predictions indicate that Kaempferol and Luteolin possess relatively balanced pharmacokinetic characteristics, whereas Stigmasterol may require formulation optimization to overcome solubility limitations. Nevertheless, these computational predictions remain preliminary and should be interpreted cautiously until confirmed through experimental pharmacokinetic and toxicological investigations [56,60].

Altogether, stigmasterol, luteolin, and kaempferol may represent potential multitarget phytochemicals capable of contributing to the management of diarrhea, microbial infections, and thrombotic complications. The molecular docking results, together with favorable pharmacokinetic predictions, suggest possible interactions with relevant biological targets, indicating their promise as polypharmacological leads. However, these computational findings should be interpreted cautiously, as docking simulations provide predictive insights rather than definitive evidence of biological activity. Although the calculated binding affinities were comparable to those of standard ligands such as ciprofloxacin, loperamide, and streptokinase in some cases, experimental validation is necessary to confirm these interactions and their pharmacological relevance. Furthermore, limitations such as poor aqueous solubility and potential efflux susceptibility—common characteristics of many polyphenolic compounds—highlight the need for formulation optimization and improved delivery strategies. Future studies involving bioassay-guided fractionation, target-specific biochemical assays, and expanded in vivo investigations will be essential to clarify the therapeutic potential of these phytoconstituents and to translate the present in silico predictions into practical pharmacological applications.

### Limitations and future prospective

Although this study provides compelling evidence of the pharmacological potential of the petroleum ether soluble fraction of *Eichhornia crassipes* flowers, several limitations need to be considered before translating these findings into clinical applications. The antimicrobial and thrombolytic activities observed were moderate compared to conventional drugs, which reduces their immediate therapeutic applicability. MIC values were determined to strengthen antimicrobial evaluation; however, additional studies such as minimum bactericidal concentration (MBC), time–kill kinetics, and pathogen-specific infection models (including diarrhea-relevant strains such as *Escherichia coli*) would provide a more comprehensive assessment. The *in vivo* experiments were conducted using relatively small animal groups (n = 4 per group), consistent with preliminary pharmacological screening and ethical principles of animal use (Reduction). However, this sample size may increase the risk of Type II error. Although the observed dose-dependent reductions in diarrheal count and the magnitude of differences between treated and control groups suggest a substantial biological effect, future studies incorporating a priori power analysis (e.g., using G*Power), larger cohorts, and additional endpoints such as latent period, intestinal transit, and enteropooling assays are necessary to strengthen statistical robustness and translational relevance.

Furthermore, while the study successfully isolated five compounds, it did not employ bioassay-guided fractionation, which could have provided a clearer understanding of which specific phytochemicals were responsible for the observed biological activities. In addition, although the isolated compounds were characterized using ¹H NMR spectroscopy and comparison with published data, more comprehensive structural elucidation techniques (e.g., ¹³C NMR, HMBC, HSQC, or HRMS) and quantitative purity assessment (e.g., HPLC-DAD profiling) were not included, as the present study was primarily designed as an exploratory phytochemical and pharmacological investigation focused on compound isolation and preliminary activity evaluation. In addition, the limited yield of certain isolates constrained extensive analytical characterization without compromising sample availability.

Another limitation lies in the reliance on in silico docking and ADMET predictions, which, though valuable, remain predictive tools and cannot replace detailed pharmacokinetic and pharmacodynamic evaluations in living systems. Additionally, no advanced toxicological assessment or evaluation of human clinical relevance was performed, which limits direct translational applicability of the findings. Moreover, stigmasterol, despite its strong docking performance and promising activity, demonstrated physicochemical drawbacks such as high lipophilicity and low aqueous solubility, which may limit its bioavailability and restrict its practical therapeutic use without further formulation improvements.

Looking forward, future research should address these limitations through more targeted experimental approaches. Bioassay-guided fractionation combined with advanced analytical techniques would allow clearer identification of the specific compounds responsible for the observed activities. Comprehensive metabolite profiling using chromatographic techniques such as HPLC-DAD or LC–MS is also warranted to quantify relative abundance and confirm purity of the isolated constituents. In addition, mechanistic validation studies, including enzyme inhibition assays, receptor-binding experiments, and disease-relevant animal models, are necessary to confirm the biological targets suggested by docking analyses. Expanding in vivo investigations with larger animal cohorts and improved statistical power would also strengthen the reliability of pharmacological findings. Additionally, incorporation of adequately powered study designs and refined pharmacological endpoints will enhance the robustness of future evaluations. Given the physicochemical limitations observed for some compounds, particularly stigmasterol, formulation strategies such as nanoemulsions, liposomal delivery systems, or other nanocarrier-based approaches may improve solubility and bioavailability. Furthermore, synergy studies with conventional antimicrobial, antidiarrheal, or thrombolytic agents may reveal additive or potentiating effects that could enhance therapeutic relevance. Finally, comprehensive preclinical evaluations including pharmacokinetics, toxicity profiling, and long-term safety studies will be essential to determine whether these phytochemicals can be translated into viable therapeutic candidates.

## Conclusion

The present study demonstrates that the petroleum ether soluble fraction of *Eichhornia crassipes* flowers contains bioactive phytochemicals with diverse pharmacological potential. Phytochemical isolation led to the identification of stigmasterol, kaempferol, and luteolin, which may contribute to the biological activities observed in the experimental assays. The extract exhibited dose-dependent antidiarrheal activity *in vivo*, along with moderate antimicrobial activity against several bacterial and fungal pathogens and measurable thrombolytic activity *in vitro*. Although the thrombolytic effect was lower than that of the reference drug streptokinase, the results indicate a multifaceted pharmacological profile for this plant fraction. Complementary molecular docking analyses provided predictive insights into possible ligand–target interactions, suggesting that the identified phytochemicals may interact with proteins associated with antimicrobial, antidiarrheal, and thrombolytic pathways, including DHFR, KAS, opioid receptors, and tissue plasminogen activator. These findings highlight *E. crassipes* flowers as a potential source of multifunctional phytochemicals that may contribute to the development of plant-derived therapeutic leads. However, further studies involving bioassay-guided fractionation, mechanistic validation, pharmacokinetic evaluation, and advanced in vivo models will be necessary to fully establish the therapeutic relevance of these compounds. Overall, this study provides new insights into the pharmacological value of *E. crassipes* and supports continued exploration of this widely available aquatic plant as a potential resource for drug discovery.

## Supporting information

S1 File Meta data of *In vitro* and *In vivo* experiments. (XLSX)

S2 File Meta data of molecular docking. (RAR)

S3 File Figure: Graphical Abstract. (TIF)
