# Supplementary figures and images for: Isolation of bioactive compounds from the petroleum ether soluble fraction of Eichhornia crassipes (Mart.) Solms flowers with dual evaluation: In silico studies of isolated molecules and in vitro/in vivo activities of the extract
Source: PLoS One. 2026 Jun 8;21(6):e0351085. doi: 10.1371/journal.pone.0351085 (PMC13245757; doi:10.1371/journal.pone.0351085)

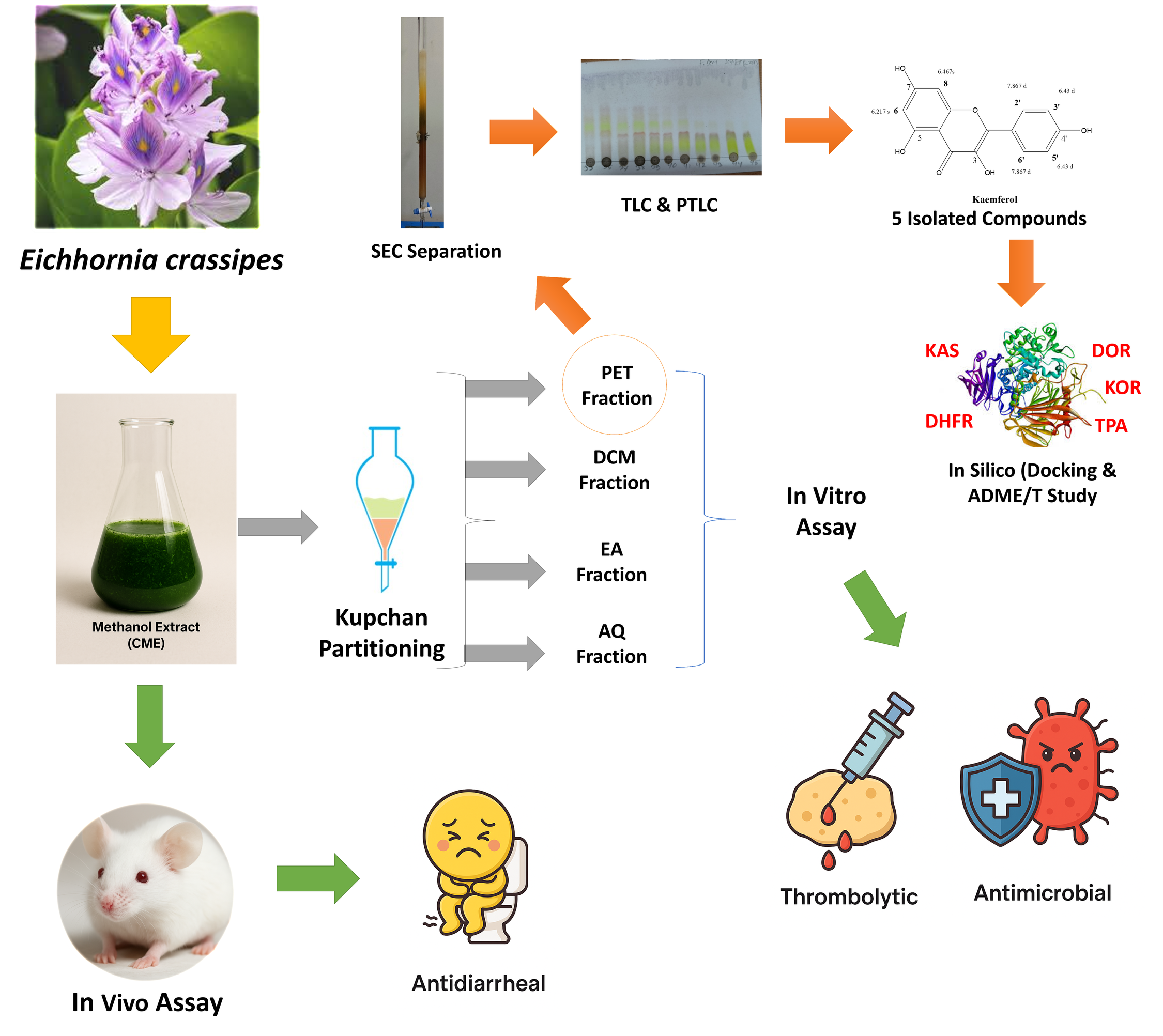

Supplement: S3 File — (TIF) [file pone.0351085.s003.tif]
